# Multiple Multi-Copper Oxidase Gene Families in Basidiomycetes – What for?

**DOI:** 10.2174/138920211795564377

**Published:** 2011-04

**Authors:** Ursula Kües, Martin Rühl

**Affiliations:** University of Goettingen, Büsgen-Institute, Division of Molecular Wood Biotechnology and Technical Mycology, Büsgenweg 2, 37077 Goettingen, Germany

**Keywords:** Agaricomycetes, ascorbate oxidase, laccase, ferroxidase, fruiting body, melanin, pigment synthesis, wood decay.

## Abstract

Genome analyses revealed in various basidiomycetes the existence of multiple genes for blue multi-copper oxidases (MCOs). Whole genomes are now available from saprotrophs, white rot and brown rot species, plant and animal pathogens and ectomycorrhizal species. Total numbers (from 1 to 17) and types of mco genes differ between analyzed species with no easy to recognize connection of gene distribution to fungal life styles. Types of mco genes might be present in one and absent in another fungus. Distinct types of genes have been multiplied at speciation in different organisms. Phylogenetic analysis defined different subfamilies of laccases sensu stricto (specific to Agaricomycetes), classical Fe2+-oxidizing Fet3-like ferroxidases, potential ferroxidases/laccases exhibiting either one or both of these enzymatic functions, enzymes clustering with pigment MCOs and putative ascorbate oxidases. Biochemically best described are laccases sensu stricto due to their proposed roles in degradation of wood, straw and plant litter and due to the large interest in these enzymes in biotechnology. However, biological functions of laccases and other MCOs are generally little addressed. Functions in substrate degradation, symbiontic and pathogenic intercations, development, pigmentation and copper homeostasis have been put forward. Evidences for biological functions are in most instances rather circumstantial by correlations of expression. Multiple factors impede research on biological functions such as difficulties of defining suitable biological systems for molecular research, the broad and overlapping substrate spectrum multi-copper oxidases usually possess, the low existent knowledge on their natural substrates, difficulties imposed by low expression or expression of multiple enzymes, and difficulties in expressing enzymes heterologously.

## INTRODUCTION

The milky sap (Urushi juice) of the lacquer tree *Rhus vernicifera* easily hardens in the presence of air. Since ancient times, this principle has been made use of in artwork in the form of lustrous translucent varnish under addition of suitable pigments, first in China and later in Japan and other Asian countries [1]. As early as in 1883, the Japanese Hikorokuro Yoshida recognized a nitrogenous heat-sensitive ‘*albuminoïd*’ in the latex of *R. vernicifera* to be the ‘*diastase*’ (the early word for enzyme) that under consumption of oxygen catalyses lacquer hardening by oxidation of urushic acid to oxo-urushic acid [2]. Within a decade, the catalyst was purified and specifically called laccase and two years later the first fungal laccases were reported [3]. Since then alone in fungi, around 100 different laccases have been purified and analyzed in biochemical properties, usually under application of substrates such as the artificial ABTS (2,2'-azino-bis(3-ethylbenzthiazoline-6-sulphonic acid)), 2,6-DMP (2,6-dimethoxyphenol) and SGZ (syringaldazine) [4,5]. In January 2011, 2227 papers in the Web of Science (Thomson Reuters) contained the word laccase in their titles. There might therefore be an impression that laccases are amongst the best understood enzymes of all. However, deeper insights reveal that we hardly know anything on the biology of the enzymes, their functions in nature and their natural substrates. Much work is driven by the high interest in laccases in biotechnology – the enzymes have multiple applications in textile industries and dye decolorization [6,7], food industries [8], wood products industries [9], paper pulp bleaching [10], bioremediation [11], biofuel cells [12], biosensor applications [13], and others [14-16]. Upon the classic review on structure and function of fungal laccases by Thurston [17], little further overview on potential biological functions can be found in the literature. Fungal laccases are suggested to act in lignocellulose degradation [18,19], in soil organic matter cycling and in ectomycorrhizal life style [20,21], in fruiting body formation [22], in different pathways of pigment production [23,24], in fungal plant-pathogen interaction [23,24] and defense [25,26], and in stress response on diverse environmental challenges [23,27] but details on the mode of actions remain unknown.

Biochemically, laccases (EC 1.10.3.2; synonyms: urishiol or urushiol oxidase, *p*-diphenol oxidase, *p*-diphenol oxidase:dioxygen oxidoreductase, benzenediol:oxygen oxidoreductase) act with low specificity on both *o-* and *p-*quinols and often also on aminophenols and phenylene-diamine. Laccases promote the following biochemical reaction under transfer of four electrons from the organic substrate to molecular oxygen:

4 benzenediol + O_2_ = 4 benzosemiquinone + 2 H_2_O.

The broad substrate spectrum impedes the definition of the enzymes. Laccases vary in substrate activity from one to another, while the full range of potential laccase substrates and especially the actual range of natural substrates remain to be identified. Moreover, laccases overlap in phenolic substrate range with tyrosinases (EC 1.14.18.1; monophenol monooxygenases, catechol-oxidases). Lack of activity on tyrosine distinguishes laccases from the latter, as well as the overall protein structure. SGZ is considered a substrate unique to laccase as long as hydrogenperoxide is avoided in enzymatic reaction [4,16,17,28].

Structurally, laccases belong to the family of blue multi-copper oxidases (MCOs) that have a three-domain structure and usually contain four copper atoms [29,30]. MCOs are characterized by the three spectroscopically different copper binding sites T1 with one copper atom (type 1 or blue, with a maximum absorbance at around 600 nm), T2 with also one copper atom (type 2 or normal, with a weak absorbance in the visible spectrum) and T3 with two copper atoms (type 3 or coupled binuclear, characterized by an absorbance at about 330 nm). T2 and T3 organize into a single copper cluster. Two histidines and one cysteine serve as ligands for copper at T1 and eight histidines for copper binding at the T2/T3 cluster. The mononuclear T1 is the primary site of electron acceptance from the substrate. Electrons are further transferred to the trinuclear cluster T2/T3 which serves as the dioxygen binding site and reduces molecular oxygen upon receipt of four electrons under formation of water [16,28,31-35]. 

**Fig. (1):**
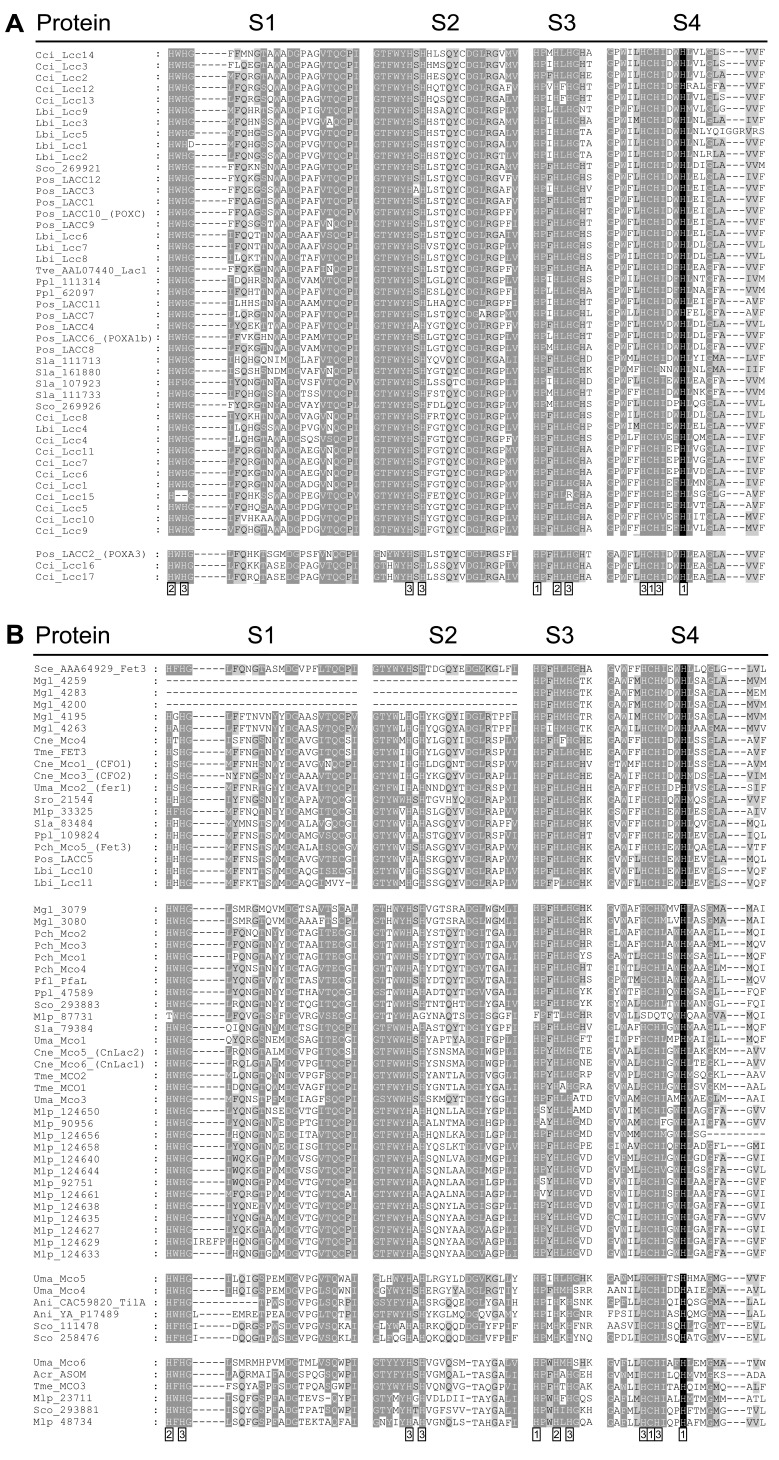
**Alignment of the sequences of the four signature motifs S1 to S4 (specifically for sensu stricto laccases L1 to L4) of fungal MCOs.** **A.** The two blocks document subfamily 1 and subfamily 2 of *sensu stricto* laccases and **B.** the blocks of sequences in series document the enzymes of the Fet3-like ferroxidase cluster, the enzymes of the ferroxidase/laccase cluster, the enzymes of the fungal pigment MCO cluster, and the enzymes of the ascorbate oxidase cluster, respectively. Enzymes follow in order the phylogenetic tree shown in Fig. (**2**). Numbers in boxes below the alignment refer to the amino acids involved in copper binding at T1, T2 or T3. Species codes: Acr, *Acremonium* sp.; Ani, *Aspergillus nidulans*; Cci, *Coprinopsis cinerea*; Cne, *Cryptococcus neoformans* var. *grubii* H99; Lbi, *Laccaria bicolor*; Mgl, *Malassezia globosa*; Mlp, *Melampsora laricis-populina*; Pch, *Phanerochaete chrysosporium*; Pfl, *Phanerochaete flavido-alba*; Pgr, *Puccinia graminis*; Ppl, *Postia placenta*; Sce, *Saccharomyces cerevisiae*; Sco, *Schizophyllum commune*; Sla, *Serpula lacrymans*; Sro, *Sporobolomyces roseus*; Tme, *Tremella mesenterica*; Tve, *Trametes versicolor*; Uma, *Ustilago maydis*. Protein codes refer either to NCBI GenBank (http://www.ncbi.nlm.nih.gov/), to protein IDs listed at the JGI portal (http://genome.jgi-psf.org/) or to names used by Hoegger *et al*. [30]. For *C. cinerea*, *L. bicolor* and *P. ostreatus*, *P. chrysosporium* and *S. commune* see [49,50,52,54,79]. Alternative names from the literature are given in brackets.

Two consensus sequences are defined for blue MCOs (PROSITE: PDOC00076; http://expasy.org/cgi-bin/get-prodoc-entry?PDOC00076): Multicopper oxidases signature 1 (PS00079) reads G-x-[FYW]-x-[LIVMFYW]-x-[CST]-x-{PR}-{K}-x(2)-{S}-x-{LFH}-G-[LM]-x(3)-[LIVMFYW] and multicopper oxidases signature 2 (PS00080) reads H-CH-x(3)-H-x(3)-[AG]-[LM]. These consensus sequences (regions) contain four (six) of the residues known to interact with copper. They overlap with L2 and L4, two of the four highly conserved sequence regions L1 to L4 that in fungal enzymes are distinguished as the fungal laccase signature sequences. L1 to L4 together cover all the copper-interacting residues [36]. Very similar highly conserved sequences are also present in other MCOs that are not classical fungal laccases. However, the corresponding regions contain specific amino acid residues that differentiate them from the conventional fungal laccases [29,37,38]. In this study, we will thus call the four conserved sequence regions with the ligands for copper binding S1 to S4 which is then generally applicable to all MCOs (Fig. **1**). 

While a broad substrate activity classifies the diphenol oxidising laccases, other MCOs can have a distinct diphenol activity: Oxidation of ascorbate to semidehydroascorbate is performed by ascorbate oxidases [38,39], oxidation of dihydroxyphenylalanine (DOPA) to dopaquinone by pigment MCOs in the DOPA pathway of melanin-synthesis [24]. Other MCOs have ferroxidase activity, i. e. they oxidize Fe^2+^ to Fe^3+ ^[40,41]. Often, such enzymes are relaxed in activity and show also degrees of classical laccase properties [40-45]. Typically, it is not clarified what might be the primary mode of biological action of such laccase-like enzymes, whether they are genuine (broad substrate) laccases also in practice or whether they are laccases with moonlighting functions [46]. Moonlighting (‘*to work at another job*’), however, in the strictest sense refers to proteins that have multiple functions within a single polypeptide chain that are not the consequence of gene fusions, splice variants, or promiscuous enzyme activities [47]. The three-dimensional organization of three-domain MCOs is highly conservative despite regions of considerable sequence divergence [48]. Stronger sequence divergence is seen particularly in regions believed to represent substrate binding domains [16,40,41,49-53]. Presence of a few specific residues in the substrate pockets of folded enzymes appears to be decisive for ferroxidase activities with negative influence on the strengths of organic compound catalysis ([40,41,53], see below). In conclusion, multi-functionalities of MCOs should therefore be better considered as promiscuous enzyme activities than representing moonligthing characters.

**Fig. (2):**
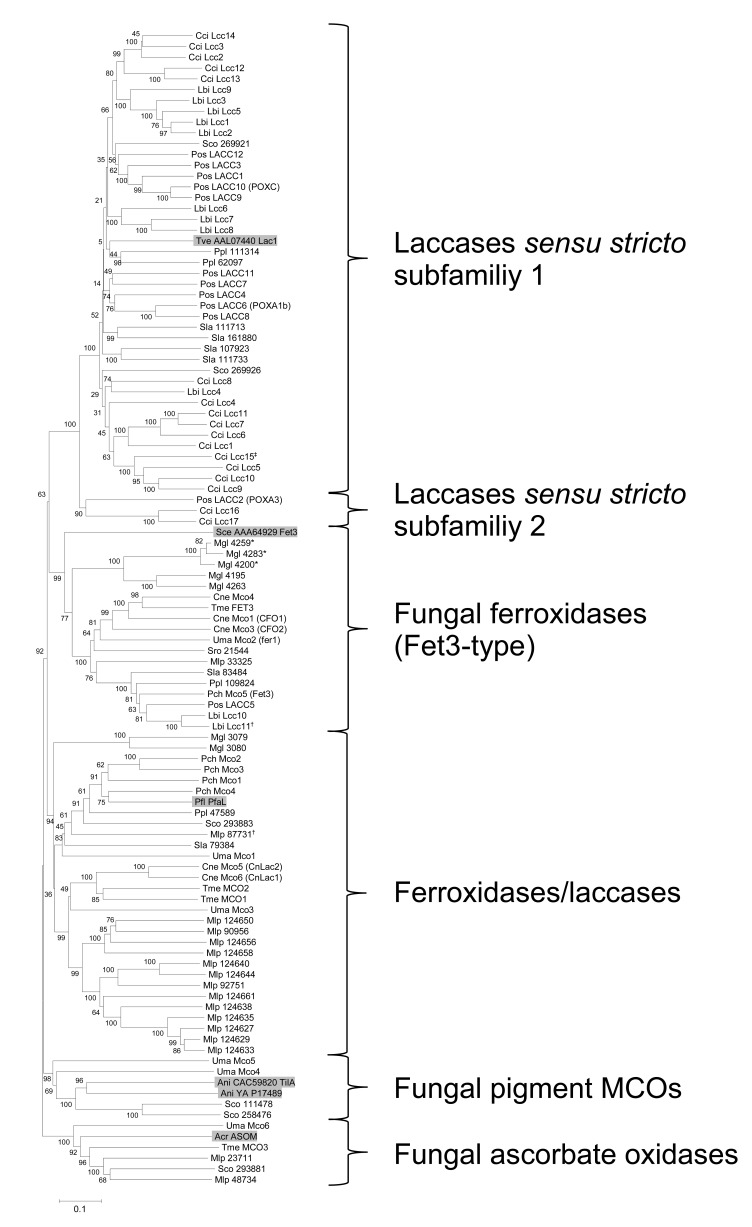
**Phylogenetic tree of aligned MCO protein sequences derived from sequenced genomes of basidiomycetes as calculated in the progam MEGA by the neighbour joining method using *p*-distance as an estimation model and pairwise deletion of the gaps.** Bootstrapping was carried out with 500 replications. For species and protein codes see legend of Fig. (**1**). * Only the C-terminal parts of the enzyme sequences are available; † one or more copper-interacting Hs are missing from a protein in the copper binding regions (see sequence alignment in Fig. **1**); ‡ Protein is incomplete by internal sequence deletion. Functionally characterized representative proteins used for defining the different MCO clusters are highlighted in grey. For better comparison to the earlier analysis performed by Hoegger *et al*. [30], the same nomenclature of enzymes are used, also for cases where in the meantime enzymes have given new specific names.

Evolutionary, all the different multi-task MCOs are related. It is possible that by evolutionary diversification, original functions of enzymes have not fully been abandoned. The phylogenetic analyses performed in this (Fig. **2**) and other studies [30,41,50,54] indicate that in fungi enzymes have undergone separate evolutionary routes towards development of untainted laccase activities (laccases *sensu stricto*, although such classical or conventional laccases may also have residual ferroxidase activity [41]) and untainted ferroxidase activities (Fet3-type ferroxidases, named after the characterized enzyme Fet3 of the ascomycete *Saccharomyces cerevisiae* [55] that however has also residual laccase activity [45]), while another evolutionary branch of enzymes contains enzymes of ostensible dual activities (ferroxidases/laccases). Within the fungi, there are also laccase-like enzymes specified on ascorbate oxidation and laccase-like enzymes specified on pigment synthesis [38,39,42,44,53,56]. In conclusion, next to the classical laccases known in multiplicity from white rot and litter decay fungi (basidiomycete laccases *sensu stricto*) and also from a number of saprotrophic and plant pathogenic filamentous ascomycetes (ascomycete laccases *sensu stricto*), at least three different lines of blue MCOs with residual laccase activity can be distinguished in fungi [30,41,50]. Only the laccases *sensu stricto* of basidiomycetes and ascomycetes seem to split from a common laccase *sensu stricto* root into two clearly distinct clusters by phylogenetic position of the two fungal phyla of dikarya [30]. Functionally, the ascomycete *sensu stricto* laccases may as the basidiomycete *sensu stricto* laccases act in lignocellulose degradation [57,58] and in oxidation of toxic phenolic compounds [59-61].

## GENES FOR BLUE MULTI-COPPER OXIDASES IN BASIDIOMYCETES

The first fungal genes for laccase-like enzymes have been detected in ascomycetes when complementing defects in spore coloration [62] or by generation of degenerate primers from partially known enzyme sequences that then were used in screening of genome libraries [63]. A similar methodology of screening a cDNA library with degenerate primers designed from known protein sequences lead to the discovery of a first conventional basidiomycete laccase gene from the white rot *Trametes hirsuta *[64]. Saloheimo *et al.* [65] obtained another basidiomycete laccase gene from a λgt11 phage library of the white rot *Phlebia radiata* cDNA in *Escherichia coli* by applying polyclonal antibodies raised against the fungal enzyme. Comparable approaches further yielded two laccase genes from the button mushroom *Agaricus bisporus* [66] and the first laccase genes from the white rots *Pleurotus ostreatus* [67] and *Trametes versicolor *[68]. With raising numbers of genes, the extreme conservation of the four protein regions with the ten histidines and the one cysteine representing the important residues within the copper-binding domains became obvious. Many more laccase genes have then been cloned from other fungal species upon designing degenerative primers from the conserved copper-binding domains. In many instances, even more than one gene per species was obtained [69-78]. The comprehensive work by Sannia and colleagues on *P. ostreatus* in summary yielded seven different genes cloned for this species [51,52,67,79-82].

The highly conserved fungal laccase signature sequences further allowed to define by tblastn searches the complete number of laccase genes in established fungal genomes (Table **1**). To surprise came that the dung fungus *Coprinopsis cinerea* has in total seventeen different genes of two different evolutionary origins [49], many more than originally anticipated for the species [74,83]. Likewise, the ectomycorrizal fungus *Laccaria bicolor* has the high number of nine laccase genes in a total of eleven *mco* genes [50] and for the white rot *P. ostreatus* twelve laccase genes were predicted [19,52], one of which however does not appear to encode a *sensu stricto* laccase (Fig. **2**). In contrast, the white-rotting weak tree pathogen *Schizophyllum commune* has only two *sensu stricto* laccase genes [84], the white rot *Phanerochaete chrysosporium *has zero [85,86] and the brown rot *Postia placenta* has also two [54]. 

When applying the sequences of the conserved copper binding domains of laccases in tblastn searches, also other *mco* genes will be detected (Figs. **1** and **2**). In addition to the above mentioned species from the Basidiomycota subphylum of the Agaricomycotina, we give here a full account of *mco* genes in the genomes of the rust *Melampsora larici-populina* of the subphylum of the Pucciniomycotina (http://genome.jgi-psf.org/Mellp1/Mellp1.home.html), of the maize pathogen *Ustilago maydis* [30,87] and the dandruff fungus *Malasezzia globosa* [88], both of the subphylum of the Ustilagomycotina, and of the saprotrophic yeast *Sporodiobolus* sp. (http://genome.jgi-psf.org/Sporo1/Sporo1.home.html; for species definition see [89]), the mycoparasitic *Tremella mesenterica* growing on wood decay fungi (http://genome.jgi-psf.org/Treme1/Treme1.home.html),**the opportunistic human pathogen* Cryptococcus neoformans* var. *grubii *[30,90], and the brown rot *Serpula lacrymans* [http://genome.jgi-psf.org/SerlaS7_3_2/SerlaS7_3_2.home.html] of the subphylum of the Agaricomycotina (Table **1**). tblastn searches were used on the released genomes to confirm previously reported genes and gene products or to obtain an account of *mco* genes for species of which whole genomes have been available for some time. Manual corrections of computer generated models were done or where required de novo models established and implemented on the genome homepages (for a full list of models analyzed, see the legend of Fig. **1**).

Numbers of *mco* genes in genomes varied between one and 17 (Table **1**). To further define the nature of genes and their products, a phylogenetic analysis of the proteins was performed by using the programs ClustalX and MEGA as described by Hoegger *et al.* [30]. MCOs of the basidiomycetes clustered into five major groups. By inclusion of characterized enzymes from other organisms [39,41,55,62,91-93], putative functions for clusters of enzymes were assigned as indicated in Table **1** and Figs. (**1** and **2**). However, as will be seen from the further discussion of the data in combination with the biological and biochemical knowledge available from the literature, this functional categorization by position in the phylogenetic tree is not utterly rigid. 

## DEFINITION OF CLUSTERS OF MCOS FROM PHYLOGENETIC ANALYSIS

The largest cluster in our analysis gave the laccase *sensu stricto* group with 45 enzymes from six different species, all of which are from the Agaricomycetes (‘*mushrooms*’). The cluster splits into two subfamilies (Fig. **2**). Within the larger subfamily 1, with the exception of the two single enzymes of *S. commune*, multiple enzymes of a species group together (Fig. **2**). As reported previously for *C. cinerea*, *L. bicolor*, and* P. ostreatus *and from enzymes of other species [30,49,54,94], the data strongly supports that genes were multiple duplicated late in evolution at the level of speciation. In accordance to an origin by recent duplication, genes for closely related laccases tend to cluster together within the genomes of Agaricomycetes [48,51,69,95], i. e. in *C. cinerea* and *P. ostreatus* in subtelomeric regions of specific chromosomes [49,96,97]. Per species, the subfamily 1 laccases divide in two or three smaller subclusters and subclusters of different species group together (Fig. **2**), suggesting that the ancient Agaricomycetes had only very few laccase genes, possibly only two or three. Enzymes of white-rots and brown-rots intermingle with enzymes from the saprotrophic *C. cinerea* and the ectomycorrhizal species *L. bicolor* (Fig. **2**). Previously, a clear differentiation between laccases from strong white-rot species and enzymes of *C. cinerea* and *L. bicolor* was seen, with laccases from the straw-decaying white-rot fungus *P. ostreatus* clustering in between [30,50,52]. Many more enzymes from different sources were included in the former analyses, especially laccases from strong wood decay species (white rots) and these likely gave rise to the much stronger separation of laccases of subfamily 1 into different groups according to substrate use (wood and straw, or organic litter) and thus life style [30]. If such tendency of laccase evolution with host substrate usages [30] holds true, what would be the targeted substrate of the ectomycorrhizal species living in symbiosis with wood plants? Indeed, supported by measured enzymatic activities and gene expression data, there is an ongoing dispute of whether ectomycorrhizal species are implemented in organic substrate decay when growing freely in the soil (see below; [50,98-102]). In this respect is also interesting that in pblast searches at NCBI (National Center for Biotechnology Information, Bethesda, MD; http://www.ncbi.nlm.nih.gov/) enzymes from the brown rot *P. placenta* best hit laccases of typical white rot basidiomycetes being active on lignin (not shown), underpinning the recently described activity of *P. placenta* laccase (Ppl_111314; Figs. **1** and **2**) on wood [103]. It remains to be noted for *P. ostreatus* that of the twelve predicted laccases [19,52], only ten cluster within subfamily 1 of *sensu stricto* laccases.

The smaller subfamily 2 of *sensu stricto* laccases with only three enzymes (Fig. **2**) contains the two closely related laccases Lcc16 and Lcc17 of *C. cinerea*. Deduced also from intron distributions, these two enzyme have an evolutionary origin different from the 15 other laccase genes of the species [49]. The third enzyme clustering with *C. cinerea* Lcc16 and Lcc17 is LACC2 (= POXA3), another laccase from *P. ostreatus* (Fig. **2**). This laccase is unusual amongst all the characterized basidiomycete laccases [4,5]. Unlike most other laccases, it acts as a heterodimer with another small protein present in two differentially glycosylated versions [80,81]. Checking the *C. cinerea* genome in tblastn searches with protein POXA3 small subunit [81] revealed no gene for an orthologous protein that could indicate presence of similar heterodimeric laccases also in *C. cinerea*. Judging from the literature, heterodimeric laccases are rare in basidiomycetes. Other than in *P. ostreatus*, heterodimeric enzymes have only been described from *A. bisporus* and from the pathogenic *Armillaria mellea* causing root and butt rot on living trees [104,105]. Former phylogenetic analyses including many more laccases from Agaricomycetes suggest that *sensu stricto* laccases may split in even more subfamilies than the two that are defined by the *C. cinerea* enzymes. When adding further enzymes, *P. ostreatus* POXA3 groups with *A. bisporus* LCC1 and LCC2 (the heterodimeric laccase described in [66,104]), separately of *C. cinerea* Lcc16 and Lcc17 which cluster with laccases from the plant pathogen *Rhizoctonia solani* and the termite symbiont *Termitomyces* sp., respectively [16,30,50,51]. The ongoing multiple projects of basidiomycete genome sequencing at JGI (Joint Genome Institute, Walnut Creek, CA) will soon shed more light into this (http://genome.jgi-psf.org/programs/fungi/index.jsf). 

In the current study within the established phylogenetic tree, the second largest group of MCOs (29 enzymes) includes known ferroxidases/laccases and the third largest group (18 enzymes) fungal ferroxidases (Fet3-type ferroxidases). Both clusters represent enzymes of species from all subphyla of the basidiomycetes, although not in all analyzed species both types of enzymes are found (Table **1**). The cluster of ferroxidases/laccases splits into two larger branches with *M. larici-populina* and *U. maydis* having representatives in both branches and into another small branch representing just two *M. globosa* MCOs (Fig. **2**). Also within the group of ferroxidase/laccase genes, duplications of genes must have happened late in evolution after speciation. Most striking is the case of *M. larici-populina* with fourteen putative genes for ferroxidases/laccases, thirteen of which cluster in the phylogenetic tree of analyzed enzymes together (Fig. **2**). *P. chrysosporium* has four closely related MCOs in the ferroxidase/laccase cluster (Fig. **2**). These enzymes come from genes located together in the fungal genome [40]. Other species have apparently only one or two genes for enzymes of putative ferroxidase/laccase activity (Fig. **2**).

Selected species from different basidiomycetes subphyla appear to harbour also genes for fungal ascorbate oxidases (the three analyzed plant pathogens and the mycoparasitic *T. mesenterica* living in wood) and two plant pathogens (*U. maydis* and *S. commune*) have also MCOs that cluster with pigment MCOs (Fig. **2**; Table **1**). Regarding the copper-binding sequences, it is interesting to note that the fungal pigment MCOs are significantly diverged from other MCOs in the S2 region and the ascorbate oxidases in the S3 and the S4 regions, respectively (Fig. **1**). The H-x-x-M-G-(M) motif in the S4 region (Fig. **1**) seems to be typical for ascorbate oxidases. In plant ascorbate oxidases, the second M in the motif serves as a fourth copper ligand in T1 [28]. The M is missing in the characterized ascomycete ascorbate oxidase and it is speculated whether the M preceding by two positions can take over the function [39]. However, unlike the *Acremonium* ascorbate oxidase ASOM ([38,39]; for further discussion see point 5 below), all basidiomycete enzymes in this cluster have the second M (Fig. **1**).

## FUNCTIONS OF MCOS IN BASIDIOMYCETES

**Fig. (3).**
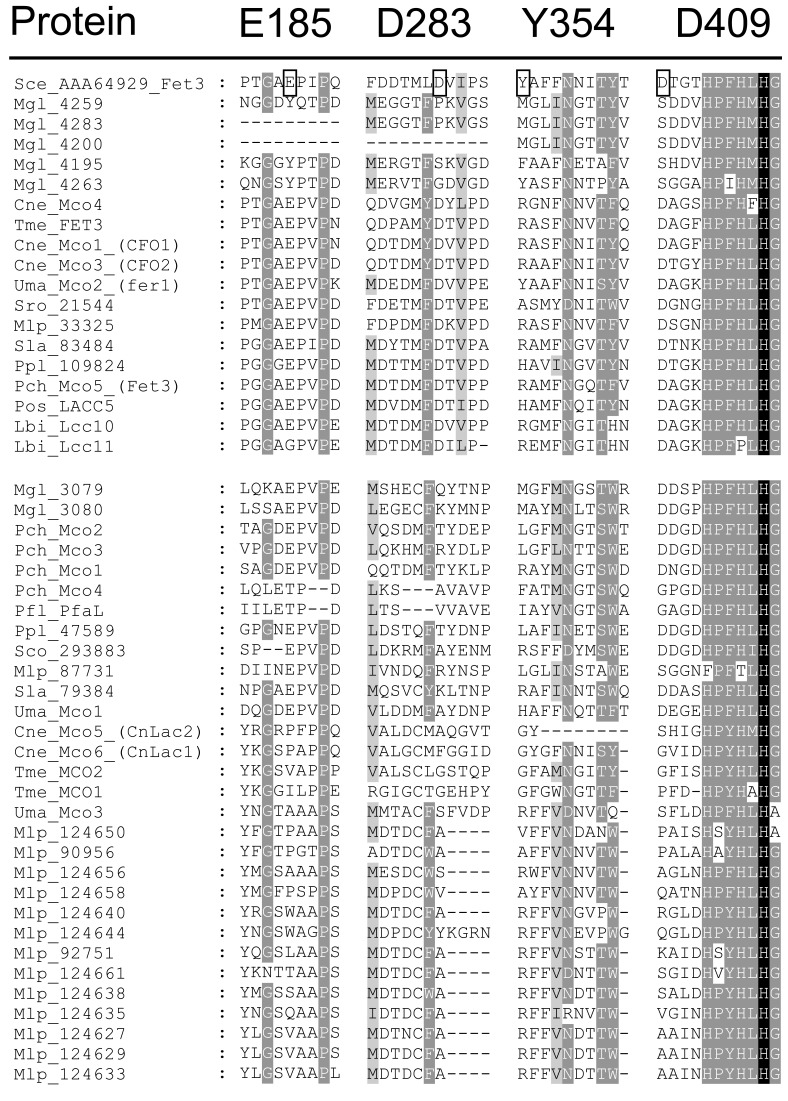
**Alignment of the four regions of the *S. cerevisiae* ferroxidase Fet3 with the residues E185, D283, Y354, D409 (boxed) known to function in oxidation of Fe^2+^ to Fe^3+^ with corresponding sequence regions of MCOs from basidiomycetes.** The upper block presents the cluster of Fet3-like enzymes and the lower block the cluster of ferroxidases/laccases following the order of the phylogenetic tree shown in Fig. (**2**).

The clustering of MCOs into distinct groups (Fig. **2**) reveals a number of interesting issues:

1. Laccases *sensu stricto* are possibly specific to the Agaricomycetes (see Fig. **2** and former phylogenetic studies [30,49,50]). Potential functions of laccases *sensu stricto* will be discussed in the following chapter.
2. The large cluster of ferroxidases/laccases contains four basidiomycete enzymes, *Cryptococcus neoformans* Mco6 (CnLac1) and Mco5 (CnLac2), *P. chrysosporium* Mco1 and *Phanerochaete flavido-alba* PfaL, of which we have some information available on enzymatic activities. The *Phanerochaete* enzymes both exhibit ferroxidase and laccase activities [40,41]. However, they differ in strength of activities: *P. chrysosporium* Mco1 has a stronger ferroxidase and a lower laccase activity than *P. flavido-alba* PfaL. Mco1 can efficiently oxidize iron and aromatic amines but not phenolic compounds [40,41]. On the contrary, PfaL indeed oxidizes a range of organic substrates including aromatic amines as well as phenols such as 2,6-DMP, gallic acid, pyrogallol, and others. PfaL oxidizes a similar substrate spectrum as a typical *sensu stricto* laccase (TvL) of the white rot *T. versicolor* and it reacts as efficiently or even better as TvL. Rodríguez-Rincón *et al.* therefore suggested to treat this enzyme as a *bona fide* laccase [41]. Specific residues (E185, D283, Y354, D409) at four different protein regions are known in *S. cerevisiae* Fet3 to contribute to catalysis of Fe^2+^. Residues E185, D283 and D409 (suggested signature motifs of multicopper ferroxidases) are decisive for Fe^2+^ oxidation whereas Y354 is less critical. E185, D283 and D409 present the binding pocket for Fe^2+^ and E185 and D409 contact Fe^2+^ and constitute parts of the electron-transfer pathway [106-111]. Importantly, *sensu stricto* laccases distinguish in these residues from ferroxidases. The acidic side chains E185, D283, and D409 in *S. cerevisiae* Fet3 confer a negatively charged surface to the mononuclear copper-binding centre T1 and mask the access of organic substrate to histidine ligands in T1 [111] as it occurs in the *sensu stricto* laccase TvL of *T. versicolor* [112]. Congruently, *P. chrysosporium* Mco1 with the stronger ferroxidase activity has two of these important residues (corresponding to E185 and D409), *P. flavido-alba* PfaL with the high laccase activity has not ([40,41]; see Fig. **3**). The comparison of the four sequence regions defined in *S. cerevisiae* Fet3 for Fe^2+^ substrate interaction may therefore help to predict which main activities enzymes within the ferroxidase/laccase cluster will exert. Most enzymes within the cluster of Fet3 ferroxidases have the three residues corresponding to E185, D283 and D409 in *S. cerevisiae* Fet3 (Fig **3**; see below point 3) and roughly one third of the enzymes in the ferroxidase/laccase cluster have residues corresponding to E185 and D409 (Fig. **3**). As stated already above, the ferroxidase/laccase cluster splits into three subclusters: In one clade reside the two *M. globosa* MCOs, both with E185 and D409 corresponding residues. In a second clade, other enzymes with also these two amino acids compile and a few exceptions of enzymes that indeed miss them. A third clade is formed exclusively from enzymes missing all the residues implicated in *S. cerevisiae* with Fet3 ferroxidase function (compare Fig. **2** and Fig. **3**). *P. flavido-alba* Pfal and *P. chrysosporium* Mco1 are found together in the second clade (Fig. **2**), indicating that the splitting within the ferroxidase/laccase cluster does not simply reflect a functional division into enzymes with a main ferroxidase activity and enzymes with a main laccase-like activity. Furthermore, *C. neoformans* Mco6 (CnLac1) found in the third subcluster (Fig. **3**) is another enzyme with comparably strong ferroxidase activity and low laccase activity [42,113] and this enzyme has none of the residues found to interact with Fe^2+^ in Fet3 of *S. cerevisiae* (Fig. **3**). Mco6 (CnLac1) is a virulence factor in *C. neoformans* [23,114-116] and Mco6 (CnLac1) localizes to the cell wall during host infection [117]. The ferroxidase activity of the enzyme appears to protect the yeast from macrophage killing by deprivation of Fe^2+^ that is converted into Fe^3+^ [42]. The multi-functional enzyme has aminophenol and polyphenol-oxidizing activities and acts in the cell wall in synthesis of heterogeneous antioxidant melanin pigments [118] by converting diphenolic or indolic substrates such as catecholamine, epinephrine, L- and D-DOPA, dopamine and caffeic acid [23,42,56,113]. The enzyme has further been shown to have prostaglandin synthase activity. Mco6 (CnLac1) converts the non-phenolic prostaglandin 2 (PGG_2_; a 20-carbon oxylipin) into prostaglandin-E_2_ (PGE_2_; a potent signalling molecule regulating inflammation in animals) and into 15-keto-PGE_2_ [119]. The closely related Mco5 (CnLac2) does not have such activity [119], although it acts on several catecholamines with different efficiencies and can replace Mco6 (CnLac1) in melanin synthesis [120]. However, the main active gene *in vivo* in infected animal hosts is *mco6* [116,120]. Mco5 (CnLac2) as Mco6 (CnLac1) is missing the four residues of *S. cerevisiae *Fet3 binding Fe^2+^ (Fig. **3**). Further to being an opportunistic human-pathogen and associated to bird excrements, *C. neoformans* occupies wounds in stems of trees as another ecological niche [121]. Further unknown tasks may be performed by the enzymes in the tannin-containing bark, the resin-secreting wounds and the decaying wood of the trees [120].
3. Most basidiomycete species contain genes for canonical Fet3-type ferroxidases (Fig. **2** and Table **1**). Fet3 as part of a high-affinity iron-uptake system of the ascomycetous yeast *S. cerevisiae* has been shown to oxidize Fe^2+^ to Fe^3+^. This step is necessary for iron uptake by a protein complex formed at the plasma membrane by Fet3 and the specific iron permease Ftr1 in order to mediate the transport of the essential metal in the Fe^3+^ form into the cells [55,106-109,122]. In accordance, *fet3* candidate genes appear often to cluster in basidiomycetes with a gene encoding a Ftr1-related potential iron permease [30]. A check for respective *ftr1 *genes in the genomes of the organisms presented in Fig. (**2**) and Table **1** confirmed such genomic arrangement for a number of the species (*U. maydis*, *P. chrysosporium*, *P. ostreatus*, *S. lacrymans*) harbouring just a single candidate *fet3* gene (this study; [30]). The *fet3* candidate gene in *P. ostreatus* coincides with one of the twelve genes previously predicted to be laccase genes [19,52]. *P. placenta* has a second copy of a gene for an iron permease (protein ID 46394) in close vicinity of a typical *fet3*-*ftr1* gene cluster [54]. *L. bicolor* has two different *fet3*-*ftr1* gene clusters [30,50], notably with the potential ferroxidase Lcc10 possessing and the potential ferroxidase Lcc11 missing the glutamic acid corresponding to the important residue E185 of *S. cerevisiae* Fet3 (Fig. **3**). Furthermore, *C. neoformans* has also two such clusters [30,123-125] and an additional gene (*mco4*) for a third putative ferroxidase without an adjacent putative iron permease gene [30]. However, a third *ftr1*-like gene is localized elsewhere in the genome (this study). In *M. larici-populina* and *T. mesenterica*, the single *fet3*- and single *ftr1*-like genes are found at separate chromosomal locations (this study). Similarly in *Sporodiobolus* sp., the single *fet3*-like gene (the sole *mco* gene of the species) and the two *ftr1*-like genes are all unlinked (this study). In the published genome of *M. globosa* [88], *ftr1*-like sequences were not found while sequences for five putative genes for MCOs clustering with Fet3-type ferroxidases (Fig. **2**) are present on very small scaffolds (sometimes only partially due to gene truncations; see Fig. **1**), suggesting that the available genome sequence is possibly not fully complete (this study). From the three genes of which enough coding sequences are available in order to deduce the regions of interest for ferroxidase activity, none of the products had the residues involved in Fe^2+^ binding in Fet3 of *S. cerevisiae *(Fig. **3**). Remarkable are further *C. cinerea* and *S. commune* that apparently have no ferroxidase genes (Table **1**) and also no *ftr1*-type iron permease genes [30; this study]. Other than using a ferroxidase/iron permease system, iron uptake might alternatively or in addition be performed by other mechanisms [122,123,126] such as a siderophore system for which for example *C. cinerea* has a respective siderophore synthesis gene [30]. Experimental evidences for ferroxidase/iron permease systems functioning in iron uptake are available from *U. maydis*,* C. neoformans* and *P. chrysosporium*. Although enzymatic ferroxidase activity was not tested, the iron permease gene *fer2* of *U. maydis* was shown to complement an iron-dependent growth defect in the orthologous gene in *S. cerevisiae*. Once either *fer2* or the ferroxidase gene *fer1* (*mco2*) were deleted from the *fer1*-*fer2* gene cluster of the plant pathogenic organism, growth on iron-limiting medium and virulence of *U. maydis* *in planta* were affected [127]. *C. neoformans* has two genes (*mco1*/*CFO1* and *mco3*/*CFO2*) that pair with adjacent iron permease genes *CFT1* and *CFT2*, respectively [30,125]. Deletion studies indicated that the *mco1* (*CFO1*)-*CFT1* gene cluster encodes the reductive high-affinity iron uptake system. The plasma-membrane-localized Mco1 (CFO1) is required for both inorganic iron utilization and full virulence, as is also the iron permease CFT1. Deletion studies of *mco3* (*CFO2*) in contrast did not result in a discernible phenotype whereas deletion of the low transcribed candidate iron transporter gene *CFT2* had however an effect on virulence [124,125]. As a further interesting phenotype, *C. neoformans mco1 *(*cfo1*) mutants show hypersensitivity to copper, indicating in addition a position for the enzyme in copper homeostasis [124]. A similar copper-sensitive phenotype has previously been described for *S. cerevisiae fet3* mutants and, moreover, Fet3 is known to oxidize also the cytotoxic Cu^1+^ to Cu^2+^ [128,129]. An alike Cu^1+^-oxidising activity by the plasma-membrane-localized Mco1 (CFO1) in *C. neoformans* could thus explain the mutant phenotype of hypersensitivity to copper [125]. In *P. chrysosporium*, expression of gene *mco5* (*fet3*) and the adjacent iron-permease gene *ftr1* is negatively iron-controlled, supporting a role of the genes in iron uptake [53]. *P. chrysosporium* Mco5 (Fet3) has a C-terminal transmembrane (TM) domain distinctive for the plasma-membrane-localized Fet3 proteins [53,55,128], differentially from the secreted ferroxidase-like enzyme Mco1 of the species (see point 2, [40]) and from the other Mcos of the species clustering in the ferroxidase/laccase cluster (Fig. **2**; [37]), all of which lack such C-terminal TM domain (this study). In the Fet3 ferroxidase cluster, sequence inspection revealed that all but the five *M. globosa* proteins have a C-terminal TM domain strongly implicating a Fet3-like function (this study). Interestingly, in other extended phylogenetic analyses including many more MCOs from ascomycetes, a group of enzymes were found to intermingle within the canonical ferroxidases that however do not have a C-terminal TM domain [30,130]. Deletion studies in the ascomycetes *Cochliobolus heterosporus* and *Aspergillus fumigatus* suggest that the respective enzymes in these organisms, ChMCO1 and Abr1, act in the DHN (1,8-dihydroxynaphthalene)-melanin synthesis pathway [130,131], one of the two known fungal pathways leading to formation of dark (brown or black) stained melanins which chemically represent a large group of diverse macromolecules formed by oxidative polymerization of phenolic or indolic compounds [24]. Inspection of the sequences of the two ascomycetes' enzymes (GenBank AB505220, AF116901) indicates that none of the four residues described in *S. cerevisiae* Fet3 for Fe^2+^ binding and catalysis are present in ChMCO1 and that only the glutamic acid corresponding to E185 of *S. cerevisiae* Fet3 is present in Abr1 (this study). Enzyme tests with SGZ documented laccase activity of the *C. heterosporus* enzyme [130]. It is suggested that such enzymes without a C-terminal TM present a new class of MCOs derived from ancient canonical ferroxidases [130]. *M. globosa* can produce melanin-like pigments from L-DOPA and cell-wall-linked phenoloxidase activities are implicated, suggesting existence of the DOPA-melanin-synthesis pathway in the dandruff fungus [132]. Functioning in melanin-synthesis rather than as canonical ferroxidases might therefore be speculated for the *M. globosa* MCOs that assembled in the phylogenetic tree in the Fet3 cluster (Fig. **2**).
4. The fourth cluster, *'fungal pigment MCOs'*, contains currently only two enzymes of *U. maydis* and two enzymes of *S. commune* (Fig. **2**). For neither, further information is available. The name of this cluster is given by *Aspergillus nidulans* YA [30], an enzyme with laccase activity that converts a yellow precursor into a green pigment which gives the *A. nidulans* conidia their typical color [44]. A related protein Abr2 from *A. fumigatus* comes from the same gene cluster for a DHN-like melanin production pathway than enzyme Abr1 discussed above (under point 3). Single gene disruptions for both genes altered spore color from bluish-green to brown, indicating that the enzymes act at different steps in pigment synthesis. Abr2 has also laccase activity and acts in conversion of a brown into a gray-green conidial pigment as part of the DHN-like melanin production pathway in this fungus [131,133]. An enzyme closely related to Abr2, *A. nidulans* TilA (Fig. **2**), has however no detectable activity in pigment production [93], indicating that a function in fungal pigment production is not granted for enzymes within this cluster. Generally, the specific reactions and the nature of the substrates of laccases and laccase-like enzymes in the DHN-melanin biosynthesis pathway need still to be defined [24].
5. So far, only one fungal ascorbate oxidase has been characterized, the thermostable enzyme ASOM from the mitosporic soil ascomycete *Acremonium* sp. and this enzyme apparently has no laccase activity [38,39]. Ascorbate oxidases are best described from plants [28] although they are not well understood in biological function. There are indications for plant ascorbate oxidases to act in oxygen homeostasis and ROS (reactive oxygen species) balancing, in various stress reactions, in defence, in growth and cell wall formation, and in signalling [134-142]. Other than in plants, genes for putative ascorbate oxidases are known from some fungi [30]. It might not be by coincidence that in this study, genes for potential ascorbate oxidases were found in all the analyzed plant pathogenic basidiomycetes (*M. larici-populina*, *U. maydis*, *S. commune*; Fig. **2**). Speculation for functioning in protection against host defence measures is therefore tempting. A similar function might be postulated for the enzyme of the mycoparasitic *T. mesenterica* living from wood decay fungi [143-145]. Interestingly, *S. commune* is also an opportunistic mycopathogen [146] and species from the ascomycete genus *Acremonium* are usually also mycoparasites [147].

## FUNCTIONS OF LACCASES *SENSU STRICTO* IN BASIDIOMYCETES

The most obvious function of laccases *sensu stricto* in the Agaricomycetes is in recalcitrant lignocellulosic substrate degradation [4,16,18,19]. Almost all white-rotting basidiomycetes produce laccases [4,5], in some cases even as the elusive and thus essential ligninolytic enzyme [84,148-150], regardless of whether performing selective or simultaneous white rot. Selective white rot is a type of decay where fungi (for example *Ceriporiopsis subvermispora*) first break down the lignin in the woody plant cell walls. In simultaneous white rot, fungi (for example *T. versicolor*) also decompose cellulose coincidently with the lignin [151-154]. In both selective and simultaneous white rot, actions of laccases are ought to attack the complex phenolic polymer lignin in the wood cell walls that, together with the hemicelluloses, mask the energy-rich cellulose for easy microbial consumption [18,155,156]. *Sensu stricto* laccases have been shown to oxidize isolated lignin under formation of phenoxy radicals [18,157]. Laccases with typical MWs in the range of 60 to 70 kDa [4,5] are however too large in size in order to easily enter themselves the intact lignified thickened secondary cell walls of limited porosity where the micropores are as small as < 6 nm in diameter [158,159]. Laccases attach to fungal cell walls of hyphae growing in wood cell lumina and to their surrounding extracellular hyphal sheaths but already early in decay laccases can be found associated with the lignin-rich middle lamella of wood cells that in selective white rot degrades first [155,156]. *In vitro*, laccases can directly act on the lignin-containing surfaces of the middle lamellae of liberated wood fibres [1,160,161] which could indicate that they may do this also at the middle lamella in the wood tissue. However, efficient degradation of lignin in the laccase-impermeable secondary cell walls must follow other means. Degradation of secondary cell walls appears to be initiated by small reactive molecules of sizes able to permeate the cell walls and act on the lignin within the secondary cell walls. Such reactive organic compounds attacking phenolic and non-phenolic side-chains of the complex lignin molecules might be contributed by laccase action due to production of low-molecular mass mediators [4,16,18]. Low-molecular mass mediators of high redox potential are defined as compounds that can oxidize other phenolic or non-phenolic molecules under transfer of electrons [3,162]. A variety of fungal metabolic products and also several degradation products obtained from oxidative lignin degradation are candidates to act *in vivo* as mediators on lignin upon their oxidation by laccases. The range of natural compounds to function upon laccase activation in lignin decomposition as potential mediators include 3-hydroxyanthranilate, 4-hydroxybenzoid acid, 4-hydroxybenzyl alcohol, phenol, aniline, vanillin, acetovanillone, methyl vanillate, syringaldehyde, acetosyringone, and *p*-coumaric acid [148,163-165]. As a further interesting observation, some *sensu stricto* laccases have been shown to convert Mn^2+^ to Mn^3+^ in the presence of pyrophosphate or malonic acids acting as chelators [166,167]. Production of semiquinone from hydrochinone with subsequent Mn^2+^ oxidation and, moreover, release of reactive H_2_O_2_ by semiquinone autoxidation has also been demonstrated [168]. Importantly, Mn^3+^-chelates are also very potent low molecular mass redox mediators that upon penetration into the cell walls participate in lignin decay [158,169,170].

Interestingly, laccase activity is sometimes also detected in brown-rots [103,171,172]. Brown-rots degrade cellulose and hemicellulose in wood while mineralizing only little of the lignin [173,174] by usage of small reactive agents including ROS that penetrate the lignin and initiate chemical damages to the lignin [175,176]. For the brown rot *P. placenta*, it has now been shown that enzyme Ppl_111314 (Fig. **2**) found in course of the genome project [54] is produced during growth on wood. It is a true *sensu stricto* laccase since it well oxidizes ABTS, 2,6-DMP and SGZ. The extracellular metabolite 2,5-dimethoxyhydroquinone (2,5-DMHQ) present in fungal infested wood is a natural substrate of the enzyme. 2,5-DMHQ is oxidized to 2,5-dimethoxy-1,4-benzoquinone (2,5-DMBQ), likely under production of the reactive H_2_O_2_ with the perhydroxyl radical (HOO˙) and the conjugate base superoxide (O_2_˙^-^) as intermediates [103]. 

Species growing on wood [77,177-180] or on straw [177,181-183] or on other complex organic plant litter [184-186] often show high laccase production on these substrates (citations give examples on such activities). In many instances, basidiomycetes produce more than one laccase on complex lignocellulosic substrate (for examples see [187-189]) as well as on artificial media (examples in [81,182,190-195]). Protein analyses in *P. ostreatus* and *C. cinerea* show that such laccase isoenzymes may come from different genes or present laccase isoforms coming from a same gene [80,81,196-198]. Alternate transcript splicing [82] or post-translational modifications such as C-terminal truncations and different patterns of glycosylation [81,82,197,199] can contribute to increasing the enzyme diversity of an organism.

In support of a functional role in substrate degradation and nutrition, many laccases are regulated by available nutrients: Often it is a combination of low nitrogen content and the nature of available nitrogen that is amongst the most important regulators ([178,181,200-205]; further reading in [5,16,26]). Usually, as expected for straw and wood as low-nitrogen containing substrates, a low nitrogen content favours laccase production [200,202-205] but opposite effects have sometimes also been reported [178,181,201]. Furthermore, available carbon can also play a positive or a negative role (examples are found in [202,204,206-208]; for review see [5,26]). In summary, a combination of multiple factors including nutritional conditions will decide upon laccase production [5,16,26]. Manifold literature exists on induction of laccase activities [5,26]. Copper (and other bivalent metal ions) and aromatic compounds as apparent laccase substrates can act in induction (for examples see [81,190-192,203,208-214], for review [5,26]). Particularly also addition of lignocellulosic residues can improve laccase production levels (examples [193,215,216], further reading in [26]).

Importantly, induction can be selective on different laccase genes of a species [191,209,210,217,218]. In promoters of the *P. ostreatus* *lacc10* (*poxc*) and *lacc6* (*poxA1b*) genes, typical metal response elements (MRE; consensus sequence TGCPuCXC) exist in multiplicity and most have been described functional in protein binding [219]. Other putative regulator sequences found in differential distribution in some but not all promoters of *Pleurotus *sp. laccase genes include xenobiotic response elements (XRE; consensus sequence TCACGC) and motifs resembling binding sites of the copper-responsive transcription factor ACE1 of *S. cerevisiae* (consensus sequence [TC(T)_4-6_GCTG]). Differential expression of genes [51,219,220] and the functional proof of an endogenous copper-dependent ACE1 transcription factor acting on the promoter of the *mco1* gene in *P. chrysosporium* [221] supports a potential usage of such sites. As documented by *mco2* of *P. chrysosporium* and for laccase genes of *T. versicolor*, a lack of these elements in promoter regions however does not exclude a positive response on gene transcription upon copper addition [209,221]. Other predicted regulatory motifs in promoters of *Pleurotus* sp. laccase genes resemble nutritional regulatory elements of ascomycetes such as for MIG, CRE, NIT1 and NIT2 binding [219]. In *C. cinerea*, there is some experimental evidence from non-laccase genes that such sequences might be functional [222]. However, in promoters of *C. cinerea* laccase genes, such potential nutritional regulatory elements are on the whole rare or missing [49,76]. Promoter functions in basidiomycetes are overall poorly understood by a general lack of suitable experimental studies [223]. Noticeably, promoters of the laccase genes in *C. cinerea* differ very much from each other in sequence. Simple sequence comparisons between the promoters do thus not allow definition of any potential consensus response elements [49,76]. Little is currently known on regulation of expression of laccases during growth in *C. cinerea* mycelium but that there is a dependency on medium and temperature [122,224].

A wide range of organic compounds has been shown to be oxidized by *sensu stricto* laccases of Agaricomycetes. However, catalytic constants of individual enzymes have at best been determined for a few substrates. Usually, one or more of the three typical laccase test substrates ABTS, 2,6-DMP, and SGZ or sometimes also the phenolic substrate guaiacol are applied in deeper enzyme characterization. Available enzymatic data indicate a broad range of catalytic differences between different *sensu stricto* laccases (see the extensive data compilations from literature in [4,5]). In *P. ostreatus*, LACC10 (POXC), LACC6 (POXA1b) and the heterodimeric LACC2 (POXA3) are main enzymes expressed during growth on solid and in liquid medium and they might thus participate in substrate utilization [191,225,226]. The purified enzymes differ in substrate specificities and, in magnitudes, in Km and kcat values as well as in stabilities and in pH and temperature optima. POXA3 consisting of the two varieties POXA3a and POXA3b formed by heterodimerization of the POXA3 large subunit as the *sensu stricto* laccase (Fig. **2**) with a differentially glycosylated smaller subunit is of all the most effective enzyme [80,81,227-229]. *Sensu stricto* laccases are further classified into three types by the residue positioned 10 amino acids downstream of the conserved cysteine in the S4 (L4) domain: class 1 (M), class 2 (L), class 3 (F) [230]. The residue at this position has an important effect on the redox potential of T1 copper at the active site [231]. Furthermore, the triad L-E-A at positions 6 to 8 downstream of the cysteine is considered important for the redox potential of an enzyme [232]. The variability at these positions in the *sensu stricto* laccases analyzed in this study is considerably high with very few possessing the predicted motif of high efficiency laccases (Fig. **1**). Importantly, the efficient enzyme POXA3b has the effective L-E-A-x-L motif in its large subunit LACC2 (POXA3) [230-232], unlike LACC10 (L-E-I-x-L) and LACC6 (L-D-L-x-F), (see Fig. **1**). There are other characterized laccases of *P. ostreatus *secreted into culture media (POXA1w, POXA2) that await assigment to specific genes. Also these enzymes have distinguished enzymatic properties. Notably, POXA1w is not a blue laccase but a white laccase that contains only one copper molecule. POXA1w is not active on guaiacol unlike other *P. ostreatus* laccases [228]. Available protein sequences allow comparison of substrate binding loops for characterized enzymes and for yet unknown laccases. Considerable differences are seen between the three known characterized *P. ostreatus* enzymes and also between the yet unknown enzymes [51]. *T. versicolor* laccase I has been crystallized with bound substrate 2,5-xylidine. Structural analysis revealed protein-ligand interactions with specific residues in the pocket formed by the substrate binding loops [112], making influences by sequence differences on enzymatic properties and substrate preferences plausible. The broad variety of *sensu stricto* laccases with diverse substrate binding loops within a species might be interpreted as adaptation on the multiple aromatic compounds a ligninolytic species is confronted with by ever changing pattern of phenolic and non-phenolic groups exposed by the complex lignin molecules during ongoing decay of straw and wood and the released lignin degradation products.

An interesting result documented in Fig. (**2**) and Table **1** is the higher number of laccase genes that are present in the ectomycorrhizal species *L. bicolor* and in the saprotrophic dung fungus *C. cinerea* compared to the straw- and wood-rotting species. The root symbiont *L. bicolor* and the dung fungus *C. cinerea* are not counted among typical wood degraders. Just *C. cinerea* may grow on wood and straw but with low decay and possibly no lignin degradation activities [224]. Basidiomycetes have generally an important position in plant litter degradation and humus formation by their ability to attack lignin. Lignin decomposition in soil includes steps of disintegration into aromatic oligo- or monomers with subsequent complete mineralization into CO_2_, H_2_O and minerals and polymerization of degradation products into humic compounds. Laccases may participate in all steps. Due to the large variety in composition of plant litter and ongoing de- and repolymerisations, litter degrading species have most likely to face up with many more phenolic and non-phenolic compounds than ligninolytic fungi living in wood. A higher diversity in laccases in organisms living in complex and heterogenous soil and compost is thus feasible [4,11,21,233]. A wide screen of wood-degrading, litter-degrading and also ectomycorrhizal basidiomycetes and a few coprophilous isolates of a broad systematic biodiversity indicated for 86% of all species laccase activities. Of 161 tested wood-decay fungi, only 36 had no laccase activity. Of 75 tested litter-degrading isolates, 60 showed laccase activity, 5 of 7 tested coprophilous species and 25 of 56 tested strains of ectomycorrhizal species [234]. A further list of more ectomycorrhizal species with phenol oxidase activities including possible laccase activities has been compilated by Burke and Cairns [20]. The results indicate that all types of life style allow laccase production, even for species that appear not to be adapted to decay of recalcitrant lignocellulose. Laccase activities have repeatedly been followed up in different soils from forests and also grassland [98,99,235-239] as well as presence of gene sequences [235,240-244] and transcripts of laccase genes [98,100,244,245] and all differ spatial-temporally. Sequences help to assign expressed laccases to saprotrophic and ectomycorrhizal species [244,245] and numerous genes from ectomycorrhizal species have been identified [240,244]. Available data clearly document that laccase activities in the soil do not come alone from saprotrophic species. Together with other enzymes possibly involved in mobilization of nutrients, laccases have also been shown to be secreted by ectomycorrhizal roots [246]. For the ectomycorrhizal basidiomycete *Suillus granulatus*, secreted laccase activities were increased upon litter addition to growing mycorrhized roots [247]. Also not indisputable ([50,98-2], see above), ectomycorrhizal species have hence been postulated to possess saprotrophic activities on organic litter in the soil. Expression of all *mco* genes in *L. bicolor* has been followed up in mycorrhiza with Douglas fir and with poplar as well as in fruiting bodies. Genes *lcc3* and *lcc8* were highly expressed in both types of ectomycorrhizae whereas *lcc9* and *lcc10* were found expressed in free living mycelium on glucose-rich medium. Gene *lcc7* increased in expression in fruiting bodies but showed marginal expression in ectomycorrhizae. The results suggest distinct functions for the genes in interaction with its hosts, in free living mycelium and in fruiting body development produced for sexual reproduction [50]. In another experiment studying fungus-host root interactions, seven different laccase genes were found expressed, with gene *lcc8* being highest expressed and genes *lcc2* and *lcc7* being not expressed in mycorrhiza [248]. While these data can not give definitive clues on the detailed functions of the enzymes in host interaction, nutrition and development, nevertheless a picture emerges on division of work of laccases during different life stages and nutritional conditions.

The soil and also composts are environments with much stronger competition by the manifold residing microbial organisms – fungi and bacteria - that others are naturally confronted with. Recent work more and more relates production of laccases also to types of microbial interactions. Microbial communities may for example help in interactive way each other in lignin degradation or species may use laccases in defense reactions by for example degrading antibiotic compounds or producing molecules helping in defense [4,25,26,249]. *P. ostreatus* for example reacts with typical soil fungi (*Trichoderma* sp.) or soil bacteria by enhanced laccase production [25,250,251], which can represent an altered isoenzyme pattern to normal laccase secretion from mycelium grown alone [252]. Likewise, *C. cinerea* produces laccase upon confrontation with certain bacteria [26] but which of the many possible ones are induced by a foreign organism is not yet determined. 

Laccase production for defence is also known amongst wood-decay fungi. This can be between individuals of one single species or between individuals of different species [25,249,253,254]. Laccase production is very local to the region of interaction [253,254]. We currently do not know whether laccases appointed in defence and/or attack are the same that are used in decay of lignocellulosic substrates. Observations on the weak white-rot *S. commune* might indicate a functional diversification. *S. commune* is very common in forests on decaying wood. In pure culture in the laboratory however, the fungus grows on wood but does only marginally decay the wood [255]. The species produces laccase [256,257] of a yellow type [16] but laccase production or laccase properties are apparently not sufficient for heavy decay. *S. commune* can behave aggressive against other Agaricomycetes and can at least for some time overgrow other decay species and thereby possibly live on the other species [146,255]. Contribution of the only two *sensu stricto* laccases and/or enzymes of the unusual set of other types of laccase-like enzymes (Fig. **2**; [84]) is tempting to speculate. In dual culture with *T. hirsuta*, *S. commune *was observed to produce a green-blue pigment in the zone of interaction [255]. Also this pigment production is a candidate phenotype for which the set of laccases and laccase-like enzymes of the organism might be responsible for, especially since two of the *S. commune* enzymes are in the fungal pigment MCO cluster (Fig. **2**).

As stated above, multiple *sensu stricto* laccases might differentially be expressed during different life stages. For example, expression of laccase genes *lcc1* and *lcc2* in *Trametes *sp. I-62 correlates with growth stages at young and older mycelial age [258]. Under defined environmental conditions when substrate has been consumed, mycelial growth is commonly followed by sexual reproduction. In *P. ostreatus*, it has repeatedly been shown that laccase activity within the substrate turns down at the developmental switch from vegetative growth to fruiting body production. Upon mushroom harvest, laccase activity within the substrates raises to highest levels that quickly drop again with the next flush of fruiting bodies [182,259-261]. In one exceptional report on *P. ostreatus*, laccase production increased further from growth at the shift to fruiting up to primordia development to then drastically go down with fruiting body maturation [262]. Also in *Agrocybe aegerita*, *Coprinellus congregatus* and *Lentinus* species, laccase activity goes down at onset of fruiting [263-266] and may rise again after fruiting [267]. In *A. bisporus*, laccase activities in the substrate are also highest directly prior to fruiting, and decline rapidly with hyphal aggregate formation. Enzyme activities fluctuate with periodic fruiting in approximately weekly cycles [268-271]. An* A. bisporus* mutant unable to fruit however continued over the time high laccase production within the mycelium [268]. In *S. commune*, laccase expression in the mycelium and fruiting is contrariwise regulated by light [257]. All these studies argue for involvement of laccase activity in mycelial growth likely on the level of nutrition but not directly in mushroom development. Comparably, a nutritional link is possibly responsible in *P. ostreatus *var*. florida* laccase mutants for failure of fruiting body formation resulting from decreased biomass production [272]. Higher biomass production upon laccase induction by veratryl alcohol explains also increase in fruiting body production and fruiting at earlier times of *P. ostreatus* growing on sugarcane bagasse-wheat bran medium [262]. In *Volvariella volvacea* in contrast, following the pattern of laccase activities in the mycelium, expression of laccase genes *lac1* (encoding a white laccase with poor oxidative activity on guaiacol) and *lac4* in the mycelium increases with time and is highest at early stages of fruiting during primordia (‘*pinhead*’) differentiation. By correlation of expression during stages of fruiting body development, functioning in fruiting has been postulated for the enzymes [75,273]. In *C. cinerea*, on nutrient-rich artificial fruiting medium laccase activity of the vegetative mycelium is negligible but laccase activity can be detected from the early beginning in developing mushrooms up to fruiting body maturation and autolysis. Expression of 15 of the in total 17 laccase genes during fruiting has been demonstrated. The pattern of expression over the time in stipe and cap tissues is very complex and currently difficult to understand [224]. Laccase activities in developing or mature fruiting bodies are also described in an ever growing list of other Agaricomycetes, such as in *Albatrella dispansum*, *Cantharellus cibarius*, *Ganoderma* species, *Pleurotus* species, *Tricholoma* *giganteum*, and *V. volvaceae* (see the compilation of references in [52]). In mature fruiting bodies of *P. ostreatus*, four different laccases were found. LACC10 (POXC), LACC6 (POYA1b) and LACC2 (POXA3) are the same as occurring during vegetative growth in the fungal mycelium, but LCC12 is a new laccase isoenzyme that is exclusively present in the fruiting bodies [52]. In *P. ostreatus* var.* florida*, the number of isoenzymes increased during sporophore development with some occuring in stipe tissues, others in the cap, and some in both. Moreover, there appears to be a natural laccase substrate in the gills [274].**In *A. bisporus*, it is in the primordia (‘*pin stage*’) where expression of gene *lcc1* increases in accordance to detected enzymatic activity [275,276] while *lcc1* and *lcc3* transcription was reported to decrease in the vegetative mycelium with onset of fruiting [95]. Further expressed in different stages of sporophore morphogenesis is laccase gene *lcc3*. This gene is also expressed in ‘*bubbles*’, masses of undifferentiated tissues produced instead of mushrooms upon fungal pathogen infection [276].

Proposed functions for laccases during mushroom development include cross-linking of cell walls of hyphae for structure stabilisation, tissue and spore pigment synthesis, and gill browning [17,22]. Conclusive experimental evidence for any of this is however not available. To our best knowledge, cross-linking of fungal cell wall and hyphal sheath compounds (mainly chitin and glucans [277] but also protein [278,279]) by laccases has not been followed up in experiments [277]. However in support of this idea, quinone as may be produced by oxidative enzymes such as laccases can react with amines on chitosan in cross-linking actions [280] and a multi-task beetle cuticle laccase (coming from an insect-specific branch of MCOs that is loosely related to the cluster of fungal pigment MCOs [30]) has now been evidenced not only to contribute to cuticle melanization but also to participate in protein-crosslinking for cuticular stiffening [281]. Some indirect data also argue for a participation of fungal laccases in pigment synthesis during steps of the fruiting process. In *Lentinula edodes*, the two genes *lac1* and *lac2* were shown to be expressed in the cap. A function in pigment synthesis, coupled to oxidative polymerization to cell wall components, was anticipated for the enzymes [282]. Furthermore, *lcc4* of *L.* *edodes* is transcribed in senescent fruiting bodies during cell wall lysis and gill browning, suggesting also a function in staining [283]. A laccase (Lcc2) isolated from fully browned fruiting bodies oxidized L-DOPA in support of a function in melanin synthesis [284]. Extracellular laccase activity has further been described in pigmented rind tissues of the cap and in stipe tissues of *L. edodes* mushrooms. A marked increase in laccase activitity in the mycelium correlated with rapid growth of pigmented primordia, suggesting also here a potential link between laccase activity and pigment production [285]. This laccase differed in size from the enzyme Lcc2 shown to oxidize L-DOPA and likely presents another enzyme [284,285]. In *C. cinerea*, the mature mushroom caps appear black due to the dark-brown spore pigmentation shining through the cap tissues [83,286]. In *C. cinerea*, there are no genes for enzymes grouping with known pigment MCOs (Fig. **2**, Table **1**; [30]). Previously, laccase activity in *C. cinerea* caps correlating well in time with coloration of spores has been reported but this enzyme does not act on L-DOPA [287]. As another possible indication of *sensu stricto* laccase contributing to melanization of fungal cell walls, growth of pigmented rhizomorphs in *Armillaria* sp. was shown to depend on expression of a laccase [288,289].

## FURTHER DETERMINATION OF FUNCTIONS OF LACCASES AND OTHER MCOS IN BASIDIOMYCETES

In conclusion from this presentation on *mco* genes in first completely available genomes of basidiomycetes, we can follow up that species very much distinguish in numbers of *mco* genes as well as in types of *mco* genes. Data may suggest that the distributions of genes in part link to life style of the species but more whole genome analyses are certainly required to substantiate any thoughts in this direction. A major problem is to know the individual functions of all the different enzymes. 

Most easy to determine are functions of genes for Fet3-like ferroxidases by heterologous expression in suitable strains of ascomycetous yeasts such as *S. cerevisiae* or *Pichia pastoris* [290]. In the dimorphic basidiomycetes *U. maydis* and *C. neoformans*, information on *mco* genes are gained by well established methods of knocking out genes and analysis of mutant phenotypes [125,126]. Such task becomes more difficult if more genes of one type are present in an organism that might have redundant functions. Moreover, knocking out genes depends on available well-working transformation methods and recombination systems [291]. For only very few species, we have functional transformation systems. In *S. commune* knocking out genes functions reasonably well [84], differentially from *C. cinerea* that has high transformation rates but very low frequencies of gene replacement by homologous recombination [292,293]. Developing specific mutants (*ku70*, *ku80*) with high frequencies of homologous recombination may solve the problem [291]. Gene silencing is an alternative that even might function *in trans* [294] but selection in case of essential or very beneficial genes might work against it. As some examples cited in the text above indicate, work-intense traditional mutagenesis yielding loss-of-function mutants or mutants with altered expression patterns might also help to get insight into specific gene functions.

Supported by annotated complete genome sequences, laccase and other MCO gene expression can be followed up by whole genome micro-array analysis – data for different species and different situations are available (for examples see [54,84,87]). Proteomic techniques can further support such large scale approaches [54,295,296]. Coincidental expression might indicate an environmental condition or a tissue in which an enzyme is active in cooperation with other functions, indicating shared physiological pathways. Most basidiomycete laccases are secreted proteins [30] but there are also reports on intracellular enzymes (for examples see [284,297,298]) and on enzymes that remain attached to the fungal cell wall and the surrounding hyphal sheaths [278,279,299-301]. Cytological studies appointing laccase-specific antibodies can thus shed further light on the identity and biology of the enzymes [119,278,299,302-304] and the search for putative natural substrates present at the same place [274]. Substrate-enzyme interactions will be a most important key to understanding the biological functions [11,20,21]. Purified enzymes are inevitably required to describe such interactions. This is not always easy to achieve. Simultaneous occurence of multiple isoenzymes and isoforms or only low expression rates of proteins or no known situations of protein expression put stress on protein isolation for individual biochemical characterization. Individual enzymes might thus functionally be characterized upon heterologous expression in selected strains of the same host of specific properties (such as a laccase-negative strain) or in heterologous fungal hosts [5,305-308]. Heterologous expression in ascomycetes is often used but not always too successful [26,308]. Successful efforts have therefore in recent time been made to overexpress laccases in homologous and heterologous basidiomycete hosts [305-307]. However, heterologous expression in another basidiomycete can sometimes be very successful [307], but sometimes also not (our unpublished observations).

In summary, while many new modern molecular, biochemical and cytological techniques are evolving in course of the large genome projects for the task to unravel specific protein functions, each individual protein in general and the various MCOs here in special will need still much trial and error work including unforseen, prior to that a biological function can definitively be assigned. It is not unlikely that larger enzymatic and biochemical networks need to be understood to then define the action(s) of an individual enzyme within.

## Figures and Tables

**Table 1:** Number of Total Potential MCOs Encoded in Genomes of Selected Basidiomycetes and their Distribution into Different Subclusters of MCOs (Compare the Phylogenetic Tree in Fig. **1**)

Species	*Melampsora larici-populina*	Ustilago maydis*	*Malassezia globosa*	*Sporobolomyces*sp.	*Tremella mesenterica*	Cryptococcus neoformans*	*Coprinopsis cinerea*	*Laccaria bicolor*	*Pleurotus ostreatus*	*Schizophyllum commune*	*Phanerochaete chrysosporium*	*Postia placenta*	*Serpula lacrymans*
Life style	Plant pathogen	Plant pathogen	Human dandruff fungus	Saprotroph	Mycoparasite living in wood	Human pathogen	Saprotroph, dung fungus	Symbiont, ectomycorrhizal species	Saprotroph, white-rot	Plant pathogen, white-rot	Saprotroph, white-rot	Saprotroph, brown-rot	Saprotroph, brown-rot
Total MCOs	17	5	7	1	4	5	17	11	12	6	5	4	6
Laccase subfamily 1	0	0	0	0	0	0	15	9	10	2	0	2	4
Laccase subfamily 2	0	0	0	0	0	0	2	0	1	0	0	0	0
Ferroxidases/ laccases	14	1	2	1	2	2	0	0	0	1	4	1	1
Fet3–type ferroxidases	1	1	5	0	1	3	0	2	1	0	1	1	1
Pigment MCOs	0	2	0	0	0	0	0	0	0	2	0	0	0
Fungal ascorbate oxidases	2	1	0	0	1	0	0	0	0	1	0	0	0

* Note that the number of mco genes is by one lower as previously been reported by Hoegger *et al*. (2006), likely due to the at the time unfinished state of the genome assemblies.
